# Association between Breakfast Consumption and Depressive Symptoms among Chinese College Students: A Cross-Sectional and Prospective Cohort Study

**DOI:** 10.3390/ijerph17051571

**Published:** 2020-02-29

**Authors:** Zhongyu Ren, Jianhua Cao, Peng Cheng, Dongzhe Shi, Bing Cao, Guang Yang, Siyu Liang, Fang Du, Nan Su, Miao Yu, Chaowei Zhang, Yaru Wang, Rui Liang, Liya Guo, Li Peng

**Affiliations:** 1 College of Physical Education, Key Laboratory of Physical Fitness Evaluation and Motor Function Monitoring, General Administration of Sport of China, Institute of Sports Science, Southwest University, Chongqing 400715, China; renzhongyu@swu.edu.cn (Z.R.); caojianhua2019@hotmail.com (J.C.); guoliya2019@sina.com (L.G.); 2 College of Physical Education, Chongqing Nursing Vocational College, Chongqing 402763, China; chengpeng777@hotmail.com; 3 Department of Physical Education, Xinhua College of Sun Yat-Sen University, Guangzhou 510520, China; shidongzhe0210@hotmail.com; 4 Key Laboratory of Cognition and Personality, Faculty of Psychology, Ministry of Education, Southwest University, Chongqing 400715, China; bingcao@swu.edu.cn; 5 School of Physical Education, Chinese Center of Exercise Epidemiology, Northeast Normal University, Changchun 130024, China; yangguang1978611@hotmail.com (G.Y.); liangsy427@nenu.edu.cn (S.L.); duf513@nenu.edu.cn (F.D.); s843505543@outlook.com (N.S.); yumiao0130@outlook.com (M.Y.); zhangcw1207@outlook.com (C.Z.); wangyr1118@outlook.com (Y.W.); Liangr0612@outlook.com (R.L.)

**Keywords:** breakfast, depressive symptoms, prospective study, college students

## Abstract

Skipping breakfast has been suggested to increase the risk of depressive symptoms, but there is no information regarding young adults. We aimed to investigate the relationship between the frequency of breakfast consumption and the risk of depressive symptoms among Chinese college students. We investigated a cross-sectional (*n* = 1060) and one-year prospective (*n* = 757) relationship between the frequency of breakfast consumption and the risk of depressive symptoms. The frequency of breakfast consumption was categorized into “≤1 time/week”, “2–5 times/week”, or “≥6 times/week”. Depressive symptoms were assessed using the 20-item Zung self-rating depression scale (SDS) with an SDS score of ≥50 to indicate moderate to severe depressive symptoms. In the cross-sectional analysis, the adjusted odds ratios (ORs) and 95% confidence intervals (CIs) of depressive symptoms related with the breakfast consumption categories were 1.00 (reference) for ≥6 times/week, 1.761 (95% CI: 1.131, 2.742) for 2–5 times/week, and 3.780 (95% CI: 1.719, 8.311) for ≤1 time/week (*p* for trend: <0.001) after adjusting for these potential confounders. Similarly, in the one-year prospective analysis, we found that 10.2% of participants was classified as having moderate to severe depressive symptoms. Multivariate logistic regressions analysis revealed a significant negative relationship between the frequency of breakfast consumption and the risk of depressive symptoms. The ORs (95% CI) for depressive symptoms with decreasing breakfast consumption frequency were 1.00 (reference) for ≥6 times/week, 2.045 (1.198, 3.491) for 2–5 times/week, and 2.722 (0.941, 7.872) for ≤1 time/week (*p* for trend: 0.005). This one-year prospective cohort study showed that skipping breakfast is related to increased risk of depressive symptoms among Chinese college students. Future research using interventional or experimental studies is required to explore the causal relationship between the effects of breakfast consumption and depressive symptoms.

## 1. Introduction

In today’s competitive world, the period of university attendance, or being a college student, is an important stage of a person’s life. College students need to adjust themselves to fit in the campus life and at the same time balance college and personal life. This possibly causes them to suffer from stress and depression. Findings from a college student-based study conducted in the U.S. reported that the prevalence of mild and severe depression was 33% [1]. Whereas in Japan, 27.8% of college freshmen reported having mild and severe depression [2]. Similarly, a meta-analysis, which included 39 studies, reported an overall prevalence of depression among Chinese university students of 23.8% [3]. Although the prevalence of depressive symptoms among college students in these studies differs from that of the general population (37.9% for depressive symptoms) [4], which may be due to the use of different measurement tools, different methodologies, and different appraisal standards, previous studies have also found a general increase in mental health problems in college students [5]. Furthermore, depression carries the greatest risk for suicide, since most suicide victims were also found to suffer from depression (60%) [6]. Previous studies also suggested that three-quarters of all lifetime cases of depression have their first onset by 24 years of age [7]. This means that depression in college students should receive more attention from public health professionals.

Breakfast has long been recognized as one of the most important meals of the day, and a healthy breakfast should contribute around 20–35% of total daily energy as it provides essential nutrient intake, which is required for the nutritional well-being of adolescents [8,9]. Furthermore, regular breakfast consumption has not only various somatic benefits, such as a lower body mass index (BMI) [10] and body fat [10], a lower risk for type 2 diabetes [11], and higher cognitive performance [12], but also mental benefits, including better overall well-being [13] and health-related quality of life [14]. Although breakfast consumption is widely considered to be an important component of a healthy lifestyle owing to its various benefits, it has been largely ignored by up to 28.9% of Chinese college students [15]. In recent years, epidemiological studies showed that long-term skipping of breakfast consumption could increase the risk of depressive symptoms, owing to an increase in cortisol levels [16,17]. Cortisol is one of the most potent anti-inflammatory hormones in the body. Higher cortisol levels are associated with an increase in inflammatory cytokines, which have negative effects on neurotransmitters by lowering serotonin and subsequently increasing the risk of depression [18]. These scientific data draw attention to the relation between breakfast consumption and depressive status.

To date, it is still unclear whether skipping breakfast could increase the risk of depressive symptoms. Most [19,20,21,22,23,24,25,26,27,28,29], but not all [30], cross-sectional studies have revealed an inverse relationship between breakfast consumption and depressive symptoms. In recent years, only three prospective cohort studies have examined this issue [31,32,33]. Two different studies revealed prospective evidence directly associating regular breakfast consumption frequency with lower risk of depressive symptoms among Japanese workers [31,33]. However, the generalization and accuracy of these two studies are limited due to failure to exclude participants with depression at baseline or sex differences (622 men and 94 women) [31,32], while another prospective study reported a failed result of the association between breakfast consumption (yes or no) and risk of depressive symptoms [33]. Furthermore, no report has so far analyzed a prospective association between breakfast consumption and depressive symptoms in young adults: e.g., college students. Adolescence is a critical time of transition since lifestyle patterns developed during this time will be carried through to adulthood.

Therefore, the aim of this study was to examine the association between breakfast consumption and the incidence of depressive symptoms among Chinese college students in a prospective setting.

## 2. Materials and Methods

### 2.1. Study Participants

Participants in this study were recruited between October 2018 and October 2019 from the Chongqing Nursing Vocational College Physical Fitness and Health cohort study, which was a prospective cohort study to assess the association between physical fitness and the health status of college students in the Chongqing Nursing Vocational College. This college is the only nursing university of Chongqing Municipality and includes 6 disciplinary categories: nursing science, midwifery, rehabilitation, aged people service and administration, community-based rehabilitation, and rehabilitation of traditional Chinese medicine.

In the first week of October 2018, we invited all 1094 college freshmen who underwent an annual physical fitness examination [lung function (forced vital capacity), physical fitness status (50 m sprint, sit and reach, standing long jump, 800/1000 m run, single-leg balance, repeated side-steps, sit-ups/pull-ups)] at the physical fitness examination center of Chongqing Nursing Vocational College in Chongqing, China. All of the 1094 college freshmen agreed to participate, and written informed consent forms were obtained from all college freshmen aged ≥16 years or from the primary guardians of participants aged <16 years. All of the participants were asked to fill out a self-designed survey questionnaire that consisted of questions about demographic (e.g., gender, age, father’s and mother’s educational level, parents’ marital status), lifestyle and health-related habits (e.g., breakfast frequency, smoking and drinking habits, and physical activity (PA), sleep duration, sleep quality), and presence of depressive symptoms. 

We excluded participants with missing information on age, height and/or body weight measurements, and PA (*n* = 34) at baseline. These exclusions gave a final cross-sectional study population of 1060 participants. For follow-up analysis, we also excluded participants with existing depressive symptoms at baseline (*n* = 108) and a further 195 individuals due to missing data on depressive symptoms during the 1-year follow-up period (the first week of October 2019). After these exclusions, this study included a total of 757 participants (693 women, 64 men; mean 18.6, standard deviation [SD] 1.0) years. Ethics approval was obtained from the Institutional Review Board of the College of Physical Education of Southwest University.

### 2.2. Assessment of Breakfast Consumption

Participants were asked to provide a response concerning breakfast consumption frequency in the lifestyle questionnaire through following question: “How often do you have breakfast in a week?” Eight response options were provided: never, 1 time/week, 2 times/week, 3 times/week, 4 times/week, 5 times/week, 6 times/week, or daily. The participants were then grouped into three categories based on their frequency of breakfast consumption: ≤1 time/week, 2–5 times/week, and ≥6 times/week.

### 2.3. Assessment of Depressive Symptoms

The Zung self-rating depression scale (SDS) was used to assess depression severity [34]. This scale consists of 20 items, and each item is scored on a scale of 1–4, with a sum score ranging from 20 to 80. High scores on the SDS represent a more severe depressive state. In the present study, an SDS score of ≥50 was used to indicate depressive symptoms.

### 2.4. Relevant Covariates

Body mass index (BMI) was calculated as weight (kg)/height^2^ (m^2^). A self-designed questionnaire [35] was used to assess demographic variables (sex, age (continuous variable), only child status (only child or nononly child), parents’ education level (senior high school or below, college, or postgraduate), and parents’ marriage status (married, widowed, or divorced)), lifestyle variables [smoking status (never, occasionally, or regularly), drinking status (never, occasionally, or regularly), sleep duration (6–8 h/day or <6 h and >8 h/day), and sleep quality (good or not good)]. PA levels were estimated using the International Physical Activity Questionnaire [36]. Total weekly PA was calculated by metabolic equivalents × hour/week [36] and categorized into 2 groups: ≥23 MET·h·week^−1^ or <23 MET·h·week^−1^ [37].

### 2.5. Statistical Analysis

Continuous variables (e.g., age or BMI) are presented as geometric least square mean (95% confidence interval) and categorical variables (e.g., sex or parents’ education level) as proportions. As age was not normally distributed, a natural logarithm transformation was run before multivariate statistical analyses. Analysis of covariance (ANCOVA) was performed for continuous variables, while multiple logistic regression analysis was performed for dichotomous variables, to test for differences in the participants’ characteristics, after adjusting for sex and age.

Depressive symptoms were used as the dependent variable and breakfast consumption as an independent variable. Multivariate logistic regression analyses were also performed to estimate the relationship between breakfast consumption and the risk of depressive symptoms. In Model 1, the analysis was conducted to calculate the crude odds ratios (OR). In Model 2, sex, age, and BMI were adjusted. Model 3 was further adjusted for other demographic variables and lifestyle factors mentioned elsewhere. In addition, *p* values of <0.05 were considered statistically significant for all two-sided tests. All tests were performed using IBM SPSS Statistics 24.0 software (IBM SPSS Inc., Chicago, IL, USA).

## 3. Results

A total of 1060 participants (103 males and 957 females) with an 18.6 ± 1.0 age took part in the study. The participants’ baseline characteristics according to category of breakfast consumption are shown in Table 1. Participants with a higher frequency of breakfast consumption demonstrated lower proportions of regular smoking and drinking (*p* = 0.005 and 0.017, respectively). Individuals with a higher frequency of breakfast consumption also demonstrated good sleep quality (*p* = 0.002). Otherwise, there were no significant differences between categories of breakfast consumption.

Out of 1060 subjects, 108 (10.2%) had depressive symptoms at baseline. Table 2 shows the relationship of breakfast consumption with prevalence of depressive symptoms at baseline. Breakfast consumption frequency was inversely associated with risk of depressive symptoms after adjustment for relevant covariates. The multivariable-adjusted ORs (95% CI) of depressive symptoms across categories of breakfast consumption was 1.00 (reference) for ≥6 times/week, 1.761 (1.131, 2.742) for 2–5 times/week, and 3.780 (1.719, 8.311) for ≤1 time/week (*p* < 0.001).

Of the 757 participants without depressive symptoms at baseline, 72 (9.5%) were newly identified as having depressive symptoms after one year. The ORs (95% CI) of depressive symptom incidence according to the baseline categories of breakfast consumption are shown in Table 3. The multivariable-adjusted ORs (95% CI) of depressive symptoms across categories of breakfast consumption was 1.00 (reference) for ≥6 times/week, 2.045 (1.198, 3.491) for 2–5 times/week and 2.722 (0.941, 7.872) for ≤1 time/week (*p* = 0.005).

## 4. Discussion

In this one-year prospective study, we examined the relationship of the frequency of breakfast consumption with the risk of depressive symptoms over time among Chinese college students. The results showed a significant relationship of breakfast omission with an increased risk of depressive symptoms after adjustment for potential confounders.

Results from this prospective cohort study support the hypothesis that a higher frequency of breakfast consumption could protect against the development of depressive symptoms. In the past, 12 different studies examined aspects related to breakfast consumption and its relation to depressive symptoms using a cross-sectional design and demonstrated a beneficial association between breakfast consumption and the risk of depressive symptoms [19,20,21,22,23,24,25,26,27,28,29]. Subsequently, several researchers conducted a Japanese worker-based prospective cohort study [31,32,33]. However, although two prospective studies on this issue revealed inverse and significant associations between breakfast consumption and the risk of depressive symptoms [31,33], the results were limited due to failure to exclude participants with depression at baseline and assessment of breakfast consumption (dichotomous variable: ≥3 times/week or not) or stratified analysis for sex (622 men and 94 women). This study was the first prospective cohort study that examined the relationship between breakfast consumption and the risk of depressive symptoms among Chinese college students.

Although the underlying biological mechanism behind the beneficial effect of breakfast consumption on depressive symptoms remains unclear, the inverse association between breakfast consumption and depressive symptoms may be explained by several plausible mechanisms that may play important roles in the pathology of depression. First, the most likely mechanism is that carbohydrate intake may mediate this relationship. Compared with irregular breakfast consumption, carbohydrate intake was significantly higher in those who consumed breakfast regularly [38]. After one night, the blood glucose concentration falls because night-time fasting causes repeated ingestion of carbohydrates [39]. To maintain normal blood glucose concentrations, the body needs to release large amounts of insulin. When blood glucose concentrations fall below normal, cortisol is released [39], which increases inflammatory cytokines [40] that negatively affect neurotransmitters by lowering serotonin, which could effectively alleviate depression [41]. In contrast, the carbohydrate supplementation after eating breakfast is able to sufficiently relieve the metabolic stress and attenuate cortisol excretion [42] with a subsequent increase in brain serotonin secretion, which is linked to positive mood [43]. Second, nutrient intake may provide another mechanism which explains the relationship between the frequency of breakfast consumption and depressive symptoms. A previous study has reported that infrequent breakfast eaters exhibited higher zinc, magnesium, and folate intake levels than habitual breakfast eaters [44]. Interestingly, several population-based meta-analyses have shown that higher zinc [45], magnesium [46], and folate intake [47] was associated with a lower risk of depressive symptoms. Unfortunately, we could not provide strong evidence for the relationship between dietary zinc, magnesium, and folate intake and the risk of depressive symptoms. Therefore, further studies are needed to assess these important nutrients. Third, in addition to biological mechanisms, socioeconomic status and daily routines could also play important roles in the association between skipping breakfast and depressive symptoms.

Population-based cross-sectional results from studies with 207,710 participants aged 20 years or over, conducted in Korea, reported that socioeconomic status, including income and educational level, biased the association between breakfast omission and depressive symptoms [28]. A previous study indicated that individuals with a higher socioeconomic status have greater motivation and resources for maintaining a healthy lifestyle [48]. Indeed, two large-scale adolescent- and adult-based cross-sectional studies also reported that individuals with a higher home income [49] and higher occupation level [50] have a higher frequency of breakfast omission. Similarly, socioeconomic status also contributes to the development of depressive symptoms [51]. Stress theory could also explain this association. Stress theory hypothesizes that personal resources, such as coping style, self-esteem, mastery, and locus of control buffer the impact of stress on depression [51,52]. Furthermore, individuals with a higher socioeconomic status are more likely to possess resources [51,52]. A meta-analysis that examined the association between socioeconomic status and the risk of depressive symptoms also supports the stress theory [51]. In addition, daily routines could also mediate the association between skipping breakfast and an increased risk of depressive symptoms. Previous studies have shown that individuals with the skipping-breakfast habit also have other health-related behaviors, including internet addiction [35], a lower physical activity level [53], and evening-type preferences [54]. Interestingly, internet addiction [55], lower physical activity level [56], and evening-type preferences [57] have been suggested to increase the risk of depressive symptoms. Unfortunately, we did not investigate the variables of socioeconomic status and daily routine. Therefore, further studies are warranted to investigate these variables.

Our study encountered several limitations that need to be addressed. First, we used the SDS, a self-reported scale, to identify depression severity (SDS score ≥50 indicates depressive symptoms), while a clinical psychiatric diagnosis would have been helpful for accurately diagnosing depression. Second, our results were limited to Chinese regional college students; hence, the existence of the above-mentioned associations in other college student populations remains unknown. Third, in the current study, we confirmed breakfast omission with an increased risk of depressive symptoms among females (*p* for trend: 0.005). For males, there was no negative association between breakfast omission and risk of depressive symptoms (*p* for trend: 0.658) (Tables S1 and S2), probably because of the small sample size. Further investigations with larger sample sizes are needed to confirm our findings. Fourth, the observational nature of the study does not allow us to establish causal relationships between breakfast consumption and depressive symptoms. Thus, future interventional or experimental research will be required to confirm this causal relationship. Fifth, exclusion of 20.5% (195/952) of participants who did not undergo a follow-up investigation might have resulted in selection bias, although the difference in baseline characteristics between the included and excluded participants was not large (see Table S3).

## 5. Conclusions

Our prospective cohort study assessed the relationship between baseline breakfast consumption and the subsequent risk of depressive symptoms in the following year among Chinese college students. The results showed a significant relationship between breakfast omission and an increased risk for depressive symptoms after adjustment for potential confounders. Findings from this study can help with the development of an effective intervention strategy against depression. Future research using interventional or experimental studies is required to explore the causal relationship between the effects of breakfast consumption and depressive symptoms.

## Figures and Tables

**Table 1:** Sex and age-adjusted subject’s baseline characteristics according to frequency of consumption of breakfast ^1^.

*N* = 1060	≤1 Time/Week (*n* = 48)	2–5 Times/Week (*n* = 444)	≥6 Times/Week (*n* = 568)	*p* for Trend ^2^
Sex (female), %	93.8	90.8	89.6	0.372
Age, years	18.6 (18.3, 18.8)	18.6 (18.5, 18.7)	18.7 (18.6, 18.7)	0.561
BMI, %				
≥30 kg/m^2^	4.2	4.5	3.0	0.277
≥25 kg/m^2^ and <30 kg/m^2^	4.2	4.7	5.3	0.616
Smoking status, %				
Occasionally	2.1	8.6	4.9	0.135
Regularly	6.3	0.9	0.5	0.005
Drinking status, %				
Occasionally	39.6	57.7	52.5	0.687
Regularly	4.2	1.4	0.4	0.017
PA, MET·h·week^−1^ (≥23), %	75.0	70.3	75.0	0.248
Sleep duration (6–8 h), %	85.4	89.0	90.7	0.164
Sleep quality (good), %	72.9	70.3	79.6	0.002
Father education, %				
Senior high school or less	89.6	91.9	92.1	0.640
College	10.4	7.9	7.6	0.568
Mother education, %				
Senior high school or less	93.8	96.4	94.7	0.481
College	6.3	3.6	5.3	0.481
Parent’s marital status, %				
Widowed	0.0	3.4	3.5	0.383
Divorced	20.8	12.6	10.6	0.063
Only one child, %	18.8	25.2	28.9	0.053

PA = physical activity; BMI = body mass index; MET = metabolic equivalent. ^1^ Continuous variables without a normal distribution were log-transformed; Continuous variables are expressed as means (95% CI) and categorical variables are expressed as percentages. ^2^ Linear trends were assessed using analysis of variance for continuous variables and Chi-square test for categorical variables.

**Table 2:** Adjusted relationships between breakfast consumption frequency and the risk of depressive symptoms at baseline ^a^.

*N* = 1060	Number of Case	Model 1 ^a^	Model 2 ^b^	Model 3 ^c^
Categories of Breakfast Consumption				
≥6 times/week (*n* = 568)	39	1.000 (reference) ^d^	1.000 (reference)	1.000 (reference)
2–5 times/week (*n* = 444)	58	2.038 (1.330, 3.122)	2.023 (1.319, 3.101)	1.761 (1.131, 2.742)
≤1 time/week (*n* = 48)	11	4.033 (1.910, 8.516)	4.001 (1.892, 8.460)	3.780 (1.719, 8.311)
*p* for trend ^e^	—	<0.001	<0.001	<0.001

^a^ Model 1: Crude. ^b^ Model 2: Adjusted for sex, age (continuous variable), BMI (≥30 kg/m^2^, ≥25 kg/m^2^ and <30 kg/m^2^ or not); ^c^ Model 3: Additionally adjusted for only one child (yes or no), father education (senior high school or less, college or undergraduate), mother education (senior high school or less, college or undergraduate), parents’ marital status (married, widowed, divorced), smoking status (regularly, occasionally, never), drinking status (regularly, occasionally, never), PA (≥23 MET·h·week^−1^ or not), sleep duration (6–8 h or not), good sleep quality (yes or no). ^d^ Adjusted data are expressed as odds ratio (95% confidence intervals). ^e^ *p* for trend were obtained using multivariate logistic regression analyses.

**Table 3:** Adjusted relationships between breakfast consumption frequency and the risk of depressive symptoms incidence during the 1-year follow-up period.

*N* = 757	Number of Case	Model 1 ^a^	Model 2 ^b^	Model 3 ^c^
Categories of Breakfast Consumption				
≥6 times = week (*n* = 425)	26	1.000 (reference) ^d^	1.000 (reference)	1.000 (reference)
2–5 times/week (*n* = 303)	41	2.401 (1.434, 4.021)	2.424 (1.446, 4.064)	2.045 (1.198, 3.491)
≤1 time/week (*n* = 29)	5	3.197 (1.128, 9.063)	3.181 (1.120, 9.034)	2.722 (0.941, 7.872)
*p* for trend ^e^	—	<0.001	<0.001	0.005

^a^ Model 1: Crude. ^b^ Model 2: Adjusted for sex, age (continuous variable), BMI (≥30 kg/m^2^, ≥25 kg/m^2^ and <30 kg/m^2^ or not); ^c^ Model 3: Additionally adjusted for only one child (yes or no), father education (senior high school or less, college or undergraduate), mother education (senior high school or less, college or undergraduate), parents’ marital status (married, widowed, divorced), smoking status (regularly, occasionally, never), drinking status (regularly, occasionally, never), PA (≥23 MET·h·week^−1^ or not), sleep duration (6–8 h or not), good sleep quality (yes or no) and depressive symptoms score at baseline. ^d^ Adjusted data are expressed as odds ratio (95% confidence intervals). ^e^ *p* for trend were obtained using multivariate logistic regression analyses.

## Supplementary Materials

The following are available online at https://www.mdpi.com/1660-4601/17/5/1571/s1, Table S1: Adjusted relationships between breakfast consumption frequency and the risk of depressive symptoms by sex at baseline, Table S2: Adjusted relationships between breakfast consumption frequency and the risk of depressive symptoms incidence by sex during the 1-year follow-up period, Table S3: Baseline characteristics of participants according to the participants included in and excluded from the analysis.
