# Supplementary material for: Association between Breakfast Consumption and Depressive Symptoms among Chinese College Students: A Cross-Sectional and Prospective Cohort Study
Source: Int J Environ Res Public Health. 2020 Feb 29;17(5):1571. doi: 10.3390/ijerph17051571 (PMC7084814; doi:10.3390/ijerph17051571)
Supplement: Supplementary file 1 [file ijerph-17-01571-s001.pdf]

Table S1. Adjusted relationships between breakfast consumption frequency and the risk of depressive symptoms by sex at baseline.

N = 1060	Number of Case	Model 1 [a]	Model 2 [b]	Model 3 [c]
Men (*n* = 103)				
Categories of breakfast consumption				
≥6 times/week (*n* = 59)	4	1.000 (reference)[d]	1.000 (reference)	1.000 (reference)
2–5 times/week (*n* = 41)	5	1.910 (0.480, 7.593)	1.955 (0.485, 7.879)	1.781 (0.366, 8.676)
≤1 time/week (*n* = 3)	0	0.000 (0.000,)	0.000 (0.000,)	0.000 (0.000,)
p for trend [e]	-	0.575	0.559	0.590
Women (*n* = 957)				
Categories of breakfast consumption				
≥6 times/week (*n* = 509)	35	1.000 (reference)[d]	1.000 (reference)	1.000 (reference)
2–5 times/week (*n* = 403)	53	2.051 (1.309, 3.212)	2.036 (1.299, 3.190)	1.735 (1.089, 2.766)
≤1 time/week (*n* = 45)	11	4.382 (2.046, 9.384)	4.361 (2.034, 9.348)	3.908 (1.739, 8.781)
p for trend [e]	-	<0.001	<0.001	0.001

[a] Model 1: Crude. [b] Model 2: Adjusted for age (continuous variable), BMI (≥30 kg/m2, ≥25 kg/m2and <30 kg/m2 or not); [c] Model 3: Additionally adjusted for only on child (yes or no), father education (senior high school or less, college or undergraduate), mother education (senior high school or less, college or undergraduate), parent's marital status (married, widowed, divorced), smoking status (regularly, occasionally, never), drinking status (regularly, occasionally, never), PA (≥23 MET·h·week^{-1} or not), sleep duration (6–8 h or not), good sleep quality (yes or no). [d] Adjusted data are expressed as odds ratio (95% confidence intervals). [e] *p* for trend were obtained using multivariate logistic regression analyses.

Table S2. Adjusted relationships between breakfast consumption frequency and the risk of depressive symptoms incidence by sex during the 1-year follow-up period.

N = 1060	Number of Case	Model 1 [a]	Model 2 [b]	Model 3 [c]
Men (*n* = 64)				
Categories of breakfast consumption				
≥6 times/week (*n* = 59)	4	1.000 (reference) [d]	1.000 (reference)	1.000 (reference)
2–5 times/week (*n* = 41)	5	2.063 (0.320, 13.313)	2.423 (0.365, 16.104)	1.828 (0.126, 26.510)
≤1 time/week (*n* = 3)	0	0.000 (0.000,)	0.000 (0.000,)	0.158 (0.000,)
p for trend [e]	-	0.632	0.548	0.658
Women (*n* = 693)				
Categories of breakfast consumption				

≥6 times/week (*n* = 509)	35	1.000 (reference)[d]	1.000 (reference)	1.000 (reference)
2–5 times/week (*n* = 403)	53	2.435 (1.424, 4.163)	2.442 (1.427, 4.179)	2.055 (1.176, 3.591)
≤1 time/week (*n* = 45)	11	3.466 (1.207, 9.956)	3.457 (1.202, 9.943)	2.958 (1.005, 8.710)
p for trend [e]	-	<0.001	<0.001	0.005

[a] Model 1: Crude. [b] Model 2: Adjusted for age (continuous variable), BMI (≥30 kg/m2, ≥25 kg/m2 and <30 kg/m2 or not); [c] Model 3: Additionally adjusted for only on child (yes or no), father education (senior high school or less, college or undergraduate), mother education (senior high school or less, college or undergraduate), parent's marital status (married, widowed, divorced), smoking status (regularly, occasionally, never), drinking status (regularly, occasionally, never), PA (≥23 MET·h·week^{-1} or not), sleep duration (6–8 h or not), good sleep quality (yes or no) and depressive symptoms score at baseline. [d] Adjusted data are expressed as odds ratio (95% confidence intervals). [e] *p* for trend were obtained using multivariate logistic regression analyses.

Table S3. Baseline characteristics of participants according to the participants included in and excluded from the analysis.

	**Excluded Participants (*n* = 195)**	**Included Participants (*n* = 757)**	***p* Value**
Sex (female), %	84.6	91.5	0.004
Age, years	18.7 (18.5, 18.8)	18.6 (18.6, 18.7)	0.877
BMI, %			
≥30 kg/m2	15.9	0.3	<0.001
≥25 kg/$m^2$and <30 kg/m^2	6.2	4.6	0.381
Smoking status, %			
Occasionally	7.7	5.7	0.297
Regularly	1.0	0.9	0.897
Drinking status, %			
Occasionally	60.0	52.4	0.060
Regularly	1.0	0.9	0.897
PA, MET·h·week^{-1} (≥23), %	70.8	74.4	0.309
Sleep duration (6–8 h), %	87.2	91.9	0.040
Sleep quality (good), %	75.9	78.9	0.371
Father education, %			
Senior high school or less	89.7	92.3	0.240
College	10.3	7.3	0.169
Mother education, %			
Senior high school or less	0.0	4.0	0.448
College	0.0	4.0	0.448
Parent's marital status, %			

Widowed	1.5	3.7	0.142
Divorced	11.8	10.7	0.662
Only one child, %	27.2	26.4	0.830
